# Improvement of solubility, dissolution, and bioavailability of phenytoin intercalated in Mg-Al layered double hydroxide

**DOI:** 10.3389/fphar.2024.1440361

**Published:** 2024-08-02

**Authors:** Rehab Anwar Bakr, Sabna Kotta, Hibah Mubarak Aldawsari, Lubna Y. Ashri, Shaimaa M. Badr-Eldin, Heba Eltahir, Sameh A. Ahmed, Yaser M. Alahmadi, Mekky Abouzied

**Affiliations:** ^1^ Department of Pharmaceutics, Faculty of Pharmacy, King Abdulaziz University, Jeddah, Saudi Arabia; ^2^ Center of Excellence for Drug Research and Pharmaceutical Industries, King Abdulaziz University, Jeddah, Saudi Arabia; ^3^ Department of Pharmaceutics, College of Pharmacy, King Saud University, Riyadh, Saudi Arabia; ^4^ Department of Pharmacology and Toxicology (Biochemistry Subdivision), College of Pharmacy, Taibah University, Medina, Saudi Arabia; ^5^ Department of Pharmacognosy and Pharmaceutical Chemistry, College of Pharmacy, Taibah University, Medina, Saudi Arabia; ^6^ Department of Pharmacy Practice, College of Pharmacy, Taibah University, Medina, Saudi Arabia

**Keywords:** phenytoin, layered double hydroxide, bioavailability, dissolution, solubility, tablet

## Abstract

Layered double hydroxides (LDHs) are highly effective drug delivery systems, owing to their capacity to intercalate or adsorb biomaterials, flexible structure, swelling property, high stability, good biocompatibility, and ease of synthesis. Phenytoin (PHT) is an antiseizure BCS (Biopharmaceutics Classification System) class II drug, presenting low aqueous solubility. Therefore, the current study aimed at increasing its solubility, dissolution, and bioavailability. PHT was intercalated to the MgAl-LDH formed *in situ* and successful intercalation to form MgAl-PHT-LDH was confirmed by FTIR, PXRD, DSC, and TGA. Examination of particle size and morphology (by photon correlation spectroscopy and electron microscopy, respectively) confirmed the formation and intercalation of nanostructured LDH. Intercalation enhanced the saturation solubility of PHT at 25°C in 0.1N HCl and phosphate buffer (pH 6.8) by 6.57 and 10.5 times respectively. The selected drug excipient powder blend for the formulation of MgAl-PHT-LDH tablets exhibited satisfactory properties in both pre-compression parameters (angle of repose, bulk density, tapped density, Carr’s index, and Hausner ratio) and tablet characteristics (weight variation, thickness, hardness, friability, content uniformity, and disintegration time). MgAl-PHT-LDH tablets showed better dissolution of PHT compared to unprocessed PHT tablets at all time points. Oral bioavailability of MgAl-PHT-LDH tablets and unprocessed PHT tablets was tested in two groups of Sprague Dawley rats based on analysis of serum levels of both forms of PHT by UPLC-ESI-MS/MS serum. MgAl-PHT-LDH tablets demonstrated a relative bioavailability of 130.15% compared to unprocessed PHT tablets, confirming a significantly higher oral bioavailability of MgAl-PHT-LDH. In conclusion, MgAl-PHT-LDH could provide a strategy for enhancing solubility, dissolution, and thereby bioavailability of PHT, enabling the evaluation of theclinical efficacy of MgAl-PHT-LDH tablets for the treatment of seizures at lower PHT doses.

## 1 Introduction

To effectively develop a new oral formulation, it’s essential to grasp the barriers impacting the drug’s bioavailability. The diminished oral bioavailability commonly observed with hydrophobic drugs primarily stems from their poor solubility. Different approaches have been developed for the oral delivery of medications in order to address the physicochemical and pharmacokinetic properties of pharmaceuticals. These strategies encompass particle size reduction, chemical modification, crystal engineering, amorphization, solvent composition adjustment, complexation, prodrug synthesis, and enhancing specific surface area, such as through solid dispersions and micronization, as well as employing drug carrier systems (Yasir et al., 2010; Hua, 2020; Alqahtani et al., 2021). Interestingly, nanostructures can significantly enhance the solubility of various substances through several mechanisms (Khan et al., 2022). Among the several reported nanostructures, layered double hydroxides (LDHs) can enhance the solubility of drugs through a process known as intercalation. Increasing specific surface area, prevention of agglomeration, and protection from degradation are some of the ways by which LDH enhances the solubility of drugs with poor water-solubility and also improve stability (Ameena Shirin et al., 2021).

LDHs (anionic clays or hydrotalcite-like compounds) are inorganic lamellar nanomaterials characterized by a two-dimensional structure. Large molecules can be accommodated by these lamellar compounds because of their high surface-to-volume ratio. Charged brucite-like layers make up LDHs, and within each layer are divalent metal ions (M^II^) octahedrally bound to six hydroxyl groups (OH-). It is possible for trivalent ions (M^III^) to take the place of some of the divalent cations. This configuration leads to the development of layers with positive charge. Water molecules and transferable anions, which include hydroxyl groups, nitrates, carbonates, and sulfates, are present within the layers and preserve the charge balance (Ameena Shirin et al., 2021). LDHs have the general formulae: [M^2+^_1-x_ M _x_^3+^ (OH) _2_] ^x+^ (A^n−^) x/n. yH_2_O] wherein M^II^ is a divalent ion, M^III^ is a trivalent ion, A^n−^ is an anion, and x is the charge density of LDH layers (Mishra et al., 2018; Ameena Shirin et al., 2021).

LDH offers numerous benefits, including cost-effectiveness, biocompatibility, minimal cytotoxicity, a straightforward production process, drug safeguarding, and improved solubility. Its exceptional biocompatibility, substantial loading capacity, and stability make LDH ideal for various applications, such as cosmetics, photoluminescence, and sensors (Mishra et al., 2018). LDHs effectively transport both drugs and genes, enhancing therapeutic outcomes for conditions like inflammation, cancer, and cardiovascular disorders (Ameena Shirin et al., 2021).

Phenytoin (PHT), also known as 5,5-diphenylhydantoin or 5,5′-diphenylimidazolidine-2,4-dione, is an anticonvulsant utilized in the management of seizures. Notably, PHT displays non-linear elimination pharmacokinetics because of saturation of metabolic enzymes (Browne and LeDuc, 1995; Patocka et al., 2020). Furthermore, PHT is a thoroughly characterized drug belonging to BCS class II, demonstrating low solubility and high permeability (Pade and Stavchansky, 1998). Thus, the oral bioavailability of PHT is limited by its solubility (Widanapathirana et al., 2015). Although various formulations of PHT may dissolve at similar rates in water, major changes in the extent of PHT release can occur when the pH of the gastrointestinal tract (pH 1–8) varies. This observation aligns with the observations that a similar dissolution in water cannot guarantee a product’s bioequivalence. One possible explanation for the decreased steady-state serum levels of PHT is inadequate drug release (Serajuddin and Jarowski, 1993).

Solid dispersions, micronization, complexation, polymer conjugation, and pH modification were among some of the earlier methods tried for enhancing PHT solubility (Stavchansky and Gowan, 1984; Dobrucki and Wojciechowska, 1992; Serajuddin and Jarowski, 1993; Latrofa et al., 2001; Widanapathirana et al., 2015). In the last few years, nanostructured delivery systems such as nanoemulsions and nanoparticles were also introduced (Sheir et al., 2022; Modi et al., 2023). However, the potentials of nanostructured LDH in enhancing the solubility and/or bioavailability of PHT have not been yet elucidated and there exists a huge research gap in this area. Fortunately, MgAl-LDH has been reported to improve solubility of drugs and was suggested to enhance the bioavailabilty of intercalated drug. The solubility of drugs such as fenbufen, camptothecin, and naproxen has been improved due to intercalation in LDH (del Arco et al., 2010; Bi et al., 2014). Furthermore, LDH was shown to dissolve in an acidic environment, resulting in the release of the intercalated bioactive drug in a molecular state conducive to absorption (Bi et al., 2014; Yu et al., 2023).

Therefore, this study evaluates the possibility of enhancing the solubility of PHT by its intercalation to MgAl-LDH. In the event of successful solubility enhancement of PHT by MgAl-LDH, the study also planned to introduce the PHT-intercalated LDH (MgAl-PHT-LDH) in the form of tablet, as one of the most convenient dosage forms and to evaluate PHT bioavailability.

## 2 Materials and methods

### 2.1 Materials

Phenytoin (PHT) and Polyvinylpyrrolidone (PVP) were procured from ACROS Organics (New Jersey, United States). Aluminum nitrate nonahydrate {Al(NO_3_)_3_ 9H_2_O} was sourced from Techno Pharmchem (India), while Magnesium nitrate hexahydrate {Mg (NO_2_)_3_ 6H_2_O} was obtained from Panreac Quimica SA (Barcelona, Spain). Sodium hydroxide (NaOH) pellets and acetone were provided by ASAGGAF Pharma HOLYLAND (Saudi Arabia). AVICEL PH-101 (microcrystalline cellulose) was acquired from WINLAB (Leicestershire, United Kingdom). Hydrochloric acid (HCl) was obtained from VWR CHEMICALS and methanol from Honeywell (Riedel-de Haёn™).

### 2.2 Methods

#### 2.2.1 Synthesis of MgAl-LDH and intercalation of PHT

**FIGURE 1:**
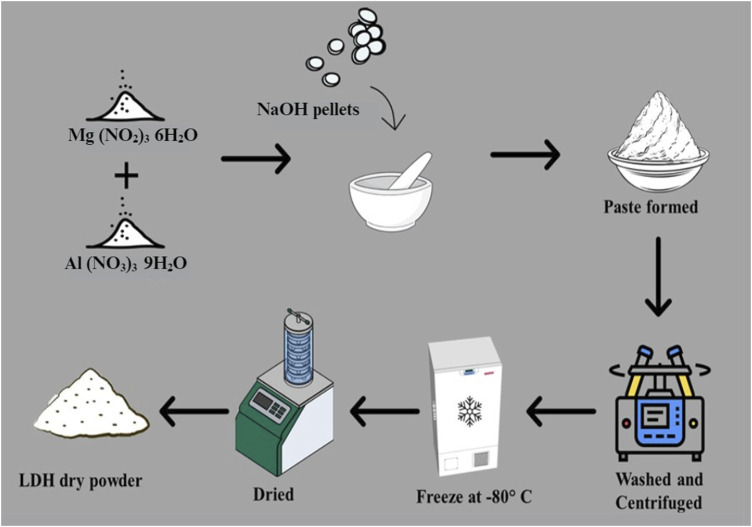
Scheme showing the synthesis of the MgAl-LDH process.

MgAl-LDH was synthesized by the trituration method, wherein 25.64 g of magnesium nitrate hexahydrate and 18.755 g of aluminium nitrate nonahydrate (molar ratio of 2:1), were combined. Subsequently, 15.995 g of NaOH pellets were slowly added until a paste was formed, maintaining the pH at around 9–10. This method, was adapted from previous studies with slight modifications (Ay et al., 2009; Khusnutdinov and Isupov, 2019). The resultant paste was washed with distilled water and subjected to centrifugation at 9,000 rpm for 5 min three times. The obtained suspension was then frozen at −80°C for 48 h and subsequently freeze-dried for 24 h and the yield (10 g of MgAl-LDH powder) was recorded. A scheme showing the preparation of the MgAl-LDH is provided in Figure 1.

PHT intercalation into MgAl-LDH was achieved by solvent technique in a 1:1 ratio according to the previously published method with slight modification. Specifically, 10 g of PHT was fully dissolved in 300 mL of acetone. Subsequently, 10 g of formulated MgAl-LDH was added to the acetone-PHT solution and mixed on a magnetic stirrer long enough until most of the acetone got evaporated. The mixture was then placed in a 60°C oven until complete acetone evaporation, resulting in the dry MgAl-PHT-LDH powder.

#### 2.2.2 Solubility studies

The saturation solubility of PHT and MgAl-PHT-LDH was assessed in 0.1 N hydrochloric acid (HCl), and phosphate buffer (pH 6.8). Surplus amounts of PHT and MgAl-PHT-LDH were separately added to 100 mL glass beakers under magnetic stirring at 25°C for 24 h. The PHT content in the solutions was estimated after appropriate dilution using a UV-visible spectrophotometer at 202 nm (Genesys 10uv, Thermo Fisher Scientific, United States).

#### 2.2.3 Characterization of PHT, MgAl-LDH and MgAl-PHT-LDH

FTIR spectra were utilized to characterize PHT and for the confirmation of the formation of MgAl-LDH, as well as the intercalation of PHT into MgAl-LDH. PXRD patterns of the PHT, MgAl-LDH, and MgAl-PHT-LDH dry powders were obtained using Bruker AXS D8 Advance. DSC thermograms of PHT, MgAl-LDH, and MgAl-PHT-LDH were obtained at 25°C–310°C, at 10°C/min using DSC60, SHIMADZU. TGAs of PHT, MgAl-LDH, and MgAl-PHT-LDH were done with an inert N_2_ atmosphere, at 10°C/min, spanning a temperature of 25°C–700°C using Perkin Elmer, Diamond TG/DTA.

The sizes of PHT, MgAl-LDH, and MgAl-PHT-LDH were determined by photon correlation spectroscopy using a Zetasizer Nano S v.7.13 instrument. SEM images of PHT, MgAl-LDH, and MgAl-PHT-LDH were captured using a JEOL Model - JSM 6390LV instrument at a 20 kV. TEM images of MgAl-PHT-LDH powder were also obtained using FEI Titan, Thermo Fisher Scientific, United States.

#### 2.2.4 Formulation of MgAl-PHT-LDH tablets

##### 2.2.4.1 Preformulation studies

Meeting acceptable flowability standards is an essential prerequisite before compression, enabling effective compression of the powder mixture. The ingredients for the preparation of MgAl-PHT-LDH tablets are listed in Table 1. Before proceeding with the compression of this powder blend, preformulation studies were carried out.

**TABLE 1:** Actual weight of the ingredients used to prepare MgAl-PHT-LDH tablets.

Excipient	Quantity for one tablet (mg)
MgAl-LDH-PHT	200
Cellulose, microcrystalline (Avicel^®^)	60
Polyvinylpyrrolidone (PVP)	9
Starch	28
Talc	1.5
Magnesium stearate	1.5

The prepared powder blends’ flow characteristics were assessed using an analysis of the Hausner ratio, Carr’s index, bulk- and tapped densities, and angle of repose. Using the fixed funnel method, which involves setting a funnel over white paper at a specific height, the angle of repose (θ) was measured. The funnel was filled with the powder sample until the pile’s top reached it. At that point, the pile’s height (h) and radius (r) were measured. Next, using the following equation, the value of θ was calculated (Eq. 1).

$$\theta =\tan^{-1}\frac{h}{r}$$ (1)

The bulk density (BD) was evaluated by weighing the powder mixture and subsequently transferring it to a measuring cylinder to estimate the volume, and finally calculated using Eq. 2.

$$Bulk\;Density=\frac{\text{Weight}\;\text{of}\;\text{the}\;\text{powder}\;\text{blend}}{\text{Bulk}\;\text{volume}\;\text{of}\;\text{the}\;\text{same}\;\text{powder}\;\text{blend}}$$ (2)

The tapped density (TD) was evaluated by weighing the powder mixture, then transferring it to a measuring cylinder placed on a wooden surface, and tapping it from a height of 1 inch either 100 times or until a consistent volume was attained. Subsequently, the TD was calculated using Eq. 3.

$$Tapped\;Density=\frac{\text{Weight}\;\text{of}\;\text{the}\;\text{powder}\;}{\text{Tapped}\;\text{volume}\;\text{of}\;\text{the}\;\text{same}\;\text{powder}\;}$$ (3)

Carr’s index and Hausner ratio were used to determine the percentage compressibility as follows (Eqs 4):

$$\text{Carr}’s\text{ index}=\left(\frac{\text{Tapped}\;\text{density}\;–\;\text{Bulk}\;\text{density}}{\text{Tapped}\;\text{density}}\right)x\;100$$ (4)

$$\text{Hausner ratio}=\frac{\text{Tapped}\;\text{density}}{\text{Bulk}\;\text{density}}$$ (5)

##### 2.2.4.2 Compaction of MgAl-PHT-LDH powder to tablets

 The details of the actual weights of the ingredients utilized in preparing the MgAl-PHT-LDH tablets and unprocessed PHT tablets are provided in Tables 1, 2 respectively. To prepare tablets, the precise amount of MgAl-PHT-LDH powder was carefully measured and combined with specified quantities of starch and PVP. Following this, blending was done after the additions of starch (disintegrant), talc (glidant), and magnesium stearate (lubricant). Avicel^®^ served as both a binder and disintegrant, while PVP acted as a binder. Subsequently, the powdered mixture was compressed into oval tablets weighing 300 mg each, containing 100 mg of PHT (n = 30), using an ERWEKA tablet machine (single punch).

**TABLE 2:** Actual weight of the ingredients used to prepare unprocessed PHT tablets.

Excipient	Quantity for one tablet (mg)
PHT	100
Cellulose microcrystalline (Avicel^®^)	60
Polyvinylpyrrolidone (PVP)	9
Starch	28
Talc	1.5
Magnesium stearate	1.5

#### 2.2.5 Evaluation of compressed tablets of MgAl-PHT-LDH

##### 2.2.5.1 Weight variation, thickness, hardness, friability, content uniformity, and disintegration time

The weight variation was determined by precisely weighing each tablet (n = 20) using an electronic balance (ADAM equipment, Maidstone Road, United Kingdom). Tablet weight was recorded as the average weight in mg ± standard deviation (SD). Additionally, ten tablets were chosen randomly and separately checked for their thickness using a Vernier caliper. The findings are presented as the mean thickness in millimeters (mm) ± SD.

For determining the tablet’s breaking strength, each tablet was placed onto the bottom plunger of a Monsanto’s hardness tester, and force was applied to break it (n = 5). The average force required, expressed in kilograms (kg), was utilized assess tablet breaking strength. The friability test (n = 10) was performed using Erweka Friabilator, where the tablets under examination were rotated within the device for 4 min at 25 rpm. Subsequently, the tablets were collected, cleaned of any dust, and weighed. The following equation (Eq. 6) was used to calculate the friability:

$$\text{Percentage friability}=\left[\frac{\left(\text{Initial}\;\text{weight}\;\text{of}\;\text{tablets}-\text{final}\;\text{weight}\;\text{of}\;\text{tablets}\right)}{\text{Initial}\;\text{weight}\;\text{of}\;\text{tablets}}\right]x\;100$$ (6)

To evaluate the content uniformity, ten tablets were individually crushed in a mortar. Subsequently 30 mL of methanol were added, mixed and the volume was made up to 100 mL with 0.1N HCL. The resultant sample was filtered and analyzed for PHT content using UV-VIS spectrophotometry by measuring the absorbance at 202 nm. The findings are presented as a percentage (%) mean ± SD.

The *in vitro* disintegration test was conducted utilizing the USP (Dst-3 automatic disintegration tester). The tablets (n = 6) were randomly selected and positioned in the mesh basket of the instrument, immersed in distilled water (37°C ± 2°C). The time taken for full disintegration with no particles visible in the mesh of the basket was taken as the disintegration time of tablet.

##### 2.2.5.2 PHT dissolution test

The dissolution test on MgAl-PHT-LDH tablets was conducted employing the USP dissolution test type II paddle apparatus (ERWEKA GmbH D-53150 Heusenstamm/Germany) at 50 rpm and 37°C ± 0.5°C. Two different dissolution media were used for the test; 0.1 N HCl (900 mL) and phosphate buffer (pH 6.8, 900 mL). Samples (5 mL) were taken at specified intervals of 5, 10, 15, 30, 60, 120, 240, and 360 min, with immediate replacement of the same volume from the dissolution media. Quantification of PHT in the withdrawn samples was achieved using UV spectrophotometry at 202 nm. The dissolution profiles were compared by calculation of similarity factor (f_2_) using the following equation (Eq. 7) (Moore and Flanner, 1996).

$$f_{2}=50•\log\;\left\{{\left[1+\left(1/n\right)\sum_{t=1}^{n}{\left(R_{t}-T_{t}\right)}^{2}\right]}^{-0.5}•100\right\}$$ (7)

##### 2.2.5.3 *In vivo* study

The study protocol was approved by the Ethics Committee for Animal Research at the college of Pharmacy, Taibah University, Saudi Arabia under reference No. COPTU-REC-73-20230694. The *in vivo* study aimed at investigating the pharmacokinetics of PHT released either from the MgAl-PHT-LDH tablets or the unprocessed PHT tablets. Six adult, male, age-mate Sprague Dawley rats (250–300 g) divided randomly into two groups (n = 3) were used. Animals were housed at standard conditions for 1 week before the experiment to acclimatize, and allowed free access to food and water. Twelve hours before starting the experimental procedures animals were fasted. Group I received MgAl-PHT-LDH tablets, while Group II received unprocessed PHT tablets. To facilitate oral administration of test drugs to the animals, tablets were dissolved in normal saline to get a final concentration of 10 mg/mL of PHT, and each animal received an oral dose of 30 mg/kg by gastric tube. Blood samples (0.5–0.6 mL) were withdrawn from the orbital sinus using a capillary tube at specified intervals (0.5, 1, 2, 3, 4, 5, 8, 12, and 24 h following dose) under light ether anesthesia. blood was allowed to clot then centrifuged to separate serum (3,000 rpm for 15 min) which was immediately frozen (−20°C) until testing for PHT concentration (Burstein et al., 1999; de Oliveira et al., 2018). The analysis of serum samples was conducted utilizing Ultra-Performance Liquid Chromatography-Electrospray Ionization-Tandem Mass Spectrometry (UPLC-ESI-MS/MS) after being de-proteinated. The detailed specifications of UPLC-ESI-MS/MS are provided in the Supplementary Data.

Non-compartmental analysis was carried out on the PHT serum concentration-time profiles. Peak serum concentration (C_max_) and time to maximum concentration (T_max_) were determined through direct examination of the graphical profiles. Linear trapezoidal summation was employed to calculate the AUC_0–24_ from time zero (pre-dose) to the last measurable serum concentration. Other pharmacokinetic parameters were also calculated and analyzed. PHT pharmacokinetic parameters following oral administration of MgAl-PHT-LDH tablets and unprocessed PHT tablets were compared using SPSS.V22, Student’s t-test with significance given at *P-value* < 0.05. Concentration *versus* time data is presented as mean ± SD.

##### 2.2.5.4 Accelerated stability studies

The study was carried out for 6 months on the prepared formulation. Tablets were placed in a desiccator at a relative humidity of 75 % ± 5%. Then the desiccator was placed in a hot air oven at 40° ± 2°C. Tablets were withdrawn from the desiccator after 6 months, and then were evaluated.

## 3 Results and discussion

### 3.1 Synthesis of MgAl-LDH and intercalation of PHT

**FIGURE 2:**
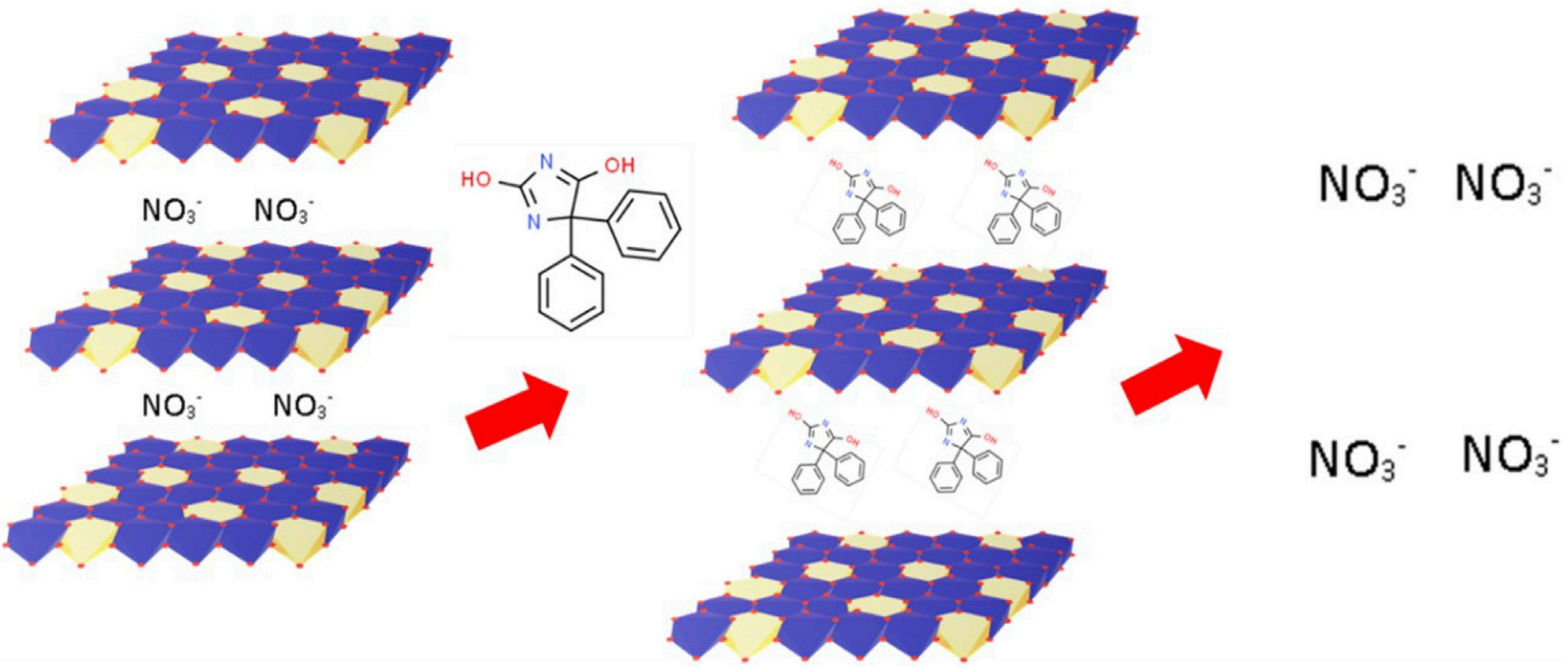
Diagram demonstrating the intercalation of PHT in the MgAl-LDH.

The MgAl-LDH synthesis process is represented schematically in Figure 1. MgAl-LDH was prepared and intercalation of PHT was done. The intercalation process of PHT in the dried MgAl-LDH is represented in Figure 2. The substitution of hydrogen ions of the nitrogen group in PHT with the positively charged MgAl-LDH layers results in the formation of a new component.

### 3.2 Solubility studies

After intercalation in the form of MgAl-PHT-LDH, the solubility of PHT at 25°C was enhanced 6.57 times in 0.1N HCl (0.4061 ± 0.001 mg/mL) and 10.5 times in pH 6.8 phosphate buffer (2.1036 ± 0.048 mg/mL) compared to unprocessed PHT, which showed solubility of 0.0618 ± 0.001 mg/mL in 0.1N HCl and 0.2 ± 0.012 mg/mL in pH 6.8 phosphate buffer. These results demonstrate the enhanced solubility of PHT upon its conversion to MgAl-PHT-LDH. The intercalation of PHT in the MgAl-LDH interlayers by the replacement of the NO_3_^−^ in the interlayer space with the PHT nitrate group could have led to the neutralization of PHT charge as well as an increase in its solubility.

Similar solubility enhancement of drugs was reported in previous studies too. A significant increase in the solubility of zaltoprofen loaded in LDH was observed compared to the drug alone, both in deionized water and in the 6.8 phosphate buffer. In water, the solubility of zaltoprofen loaded in LDH was three times higher than that of zaltoprofen alone. In the 6.8 buffer, zaltoprofen loaded in LDH showed greater solubility than zaltoprofen, exceeding eight times the solubility of the reference dose (Maggi et al., 2023). In another study, the solubility of naproxen and flurbiprofen was greatly enhanced after being intercalated with LDH. For naproxen, the solubility increased from 8 × 10^−3^ to 26 × 10^−3^ g/L after 1 min and from 30 × 10^−3^ to 146 × 10^−3^ g/L after 60 min, eventually reaching 158 × 10^−3^ g/L after 3 h. Similarly, for flurbiprofen, the solubility rose from 1 × 10^−3^ to 4 × 10^−3^ g/L after 1 min and from 11 × 10^−3^ to 56 × 10^−3^ g/L after 60 min, reaching 720 × 10^−3^ g/L after 24 h (Berber et al., 2008). These improvements are due to the drug-LDH composites’ structural properties, which prevent recrystallization and enhance hydrophilicity, leading to better water penetration and the release of the drug in an amorphous form.

### 3.3 Characterization of PHT, MgAl-LDH and MgAl-PHT-LDH

#### 3.3.1 FTIR

**FIGURE 3:**
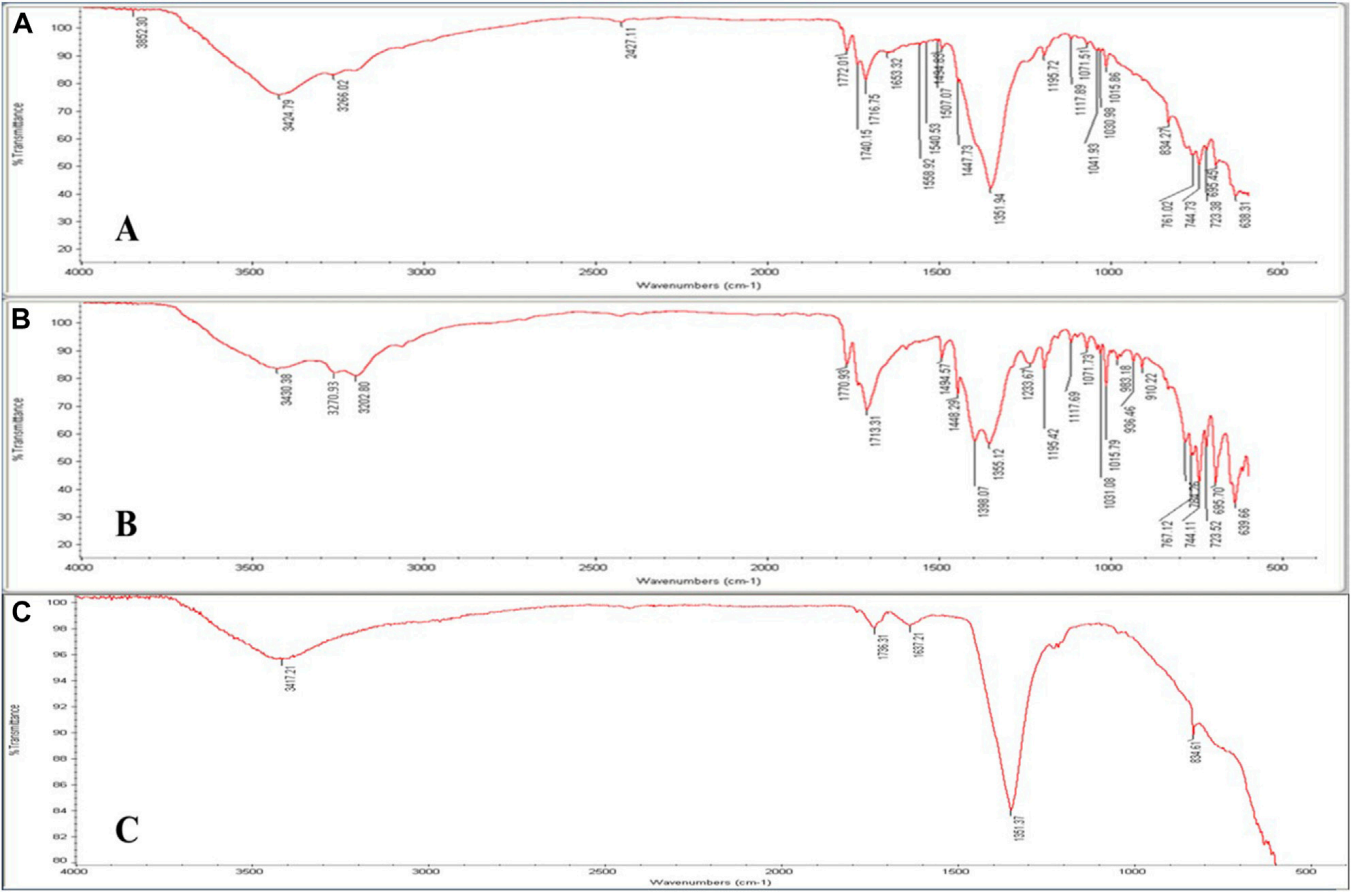
FT-IR spectra of **(A)** pure PHT, **(B)** MgAl-LDH, and **(C)** MgAl-PHT-LDH.

FTIR spectroscopy is a potent technique that may be utilized to verify drug intercalation into the LDH layers as opposed to a conventional surface adsorption. FTIR spectra of PHT, MgAl-LDH, and MgAl-PHT-LDH were obtained from 400 to 4,000 cm^–1^ (Figure 3). The spectrum of MgAl-PHT-LDH (Figure 3A) showed some differences between the pure PHT, and MgAl-LDH, which indicated the formation of a new component. Although just a few distinct functional groups appeared to be involved, the interaction appeared to encompass the whole drug molecule. The major peaks, resulting from the stretching -NH (3270.93 and 3202.80 cm^−1^) and aromatic ring (3430.38 cm^−1^) in the pure PHT spectrum were practically absent in the MgAl-PHT-LDH. The spectrum of pure PHT (Figure 3B) had distinctive peaks at 3270.93 cm^−1^ and 3202.80 cm^−1^ that refer to the NH group stretching vibrations, and at 3430.38 cm^−1^ for the -C-H stretching vibrations of aromatic molecules. Meanwhile, the peaks at 1713.31 and 1770.93 cm^−1^ indicated the carbonyl group of the structure. The peak at 1448.29 cm^−1^ indicated the C-N stretching, and those at 744.11, 723.52, and 695.70 cm^−1^ indicated the out-of-plane vibrations of the C-H bonds in the phenyl group. These results were in agreement with previous studies (Ramadhan, 2012; Suneetha et al., 2014). MgAl-LDH formation was confirmed by FTIR with the presence of a characteristic peak at 3417.21 cm^−1^ (Figure 3C) which refers to MgAl-O-H stretching, the water molecule vibrations are seen at 1637.21 cm^−1^, and a strong band at 1351.37 cm^−1^ owing to nitrate ions. This spectrum was in agreement with reported spectra displaying similar features for LDH (Ay et al., 2009).

#### 3.3.2 PXRD

**FIGURE 4:**
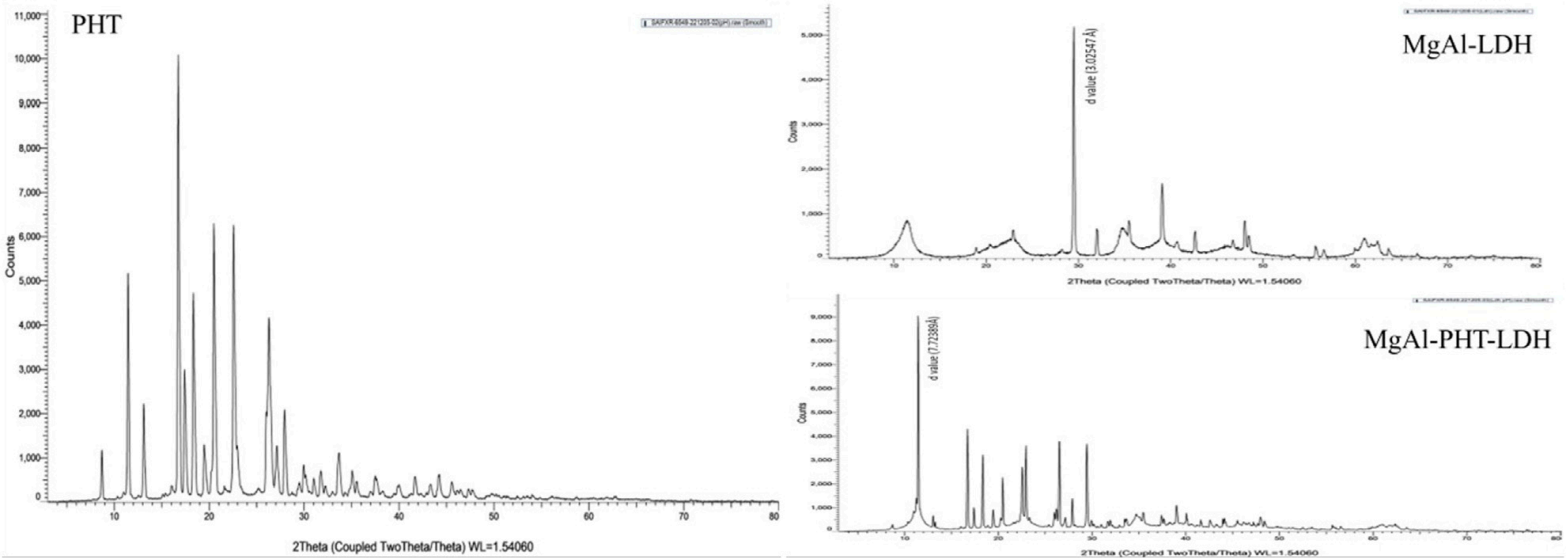
PXRD patterns of PHT, MgAl-LDH, and MgAl-PHT-LDH samples.

PXRD is an effective technique for confirming the expansion of the hydroxide layers following the replacement of counter ions with drugs. The diffraction patterns observed for PHT, MgAl-LDH, and MgAl-PHT-LDH are presented in Figure 4. The diffractogram of PHT shows sharp peaks at angles of 2θ 8.70, 11.45, 16.74, 17.41, 20.53, and 22.56°, which were consistent with a previous report (Nokhodchi et al., 2003). PXRD pattern for MgAl-LDH showed sharp and symmetric reflections, which is characteristic of crystalline nature of LDH. The basal spacing of LDH with NO3^-^ anions in the interlayer is indicated by the main diffraction peak of MgAl-LDH at 29.500° 2θ which refers to a d value of 3.02547 Å. PHT was intercalated into the MgAl-LDH interlayer, which produced a diffraction pattern characterized by a strong peak at a lower angle of 2θ° (11.447°) and an enhancement in the basal interlayer space by the substitution of PHT for the NO_3_^−^ anions. The shifting of planes to higher d values was proof of PHT intercalation into the MgAl-LDH. In MgAl-PHT-LDH, the basal spacing was raised from 3.02547 Å to 7.72389 Å. These observations were comparable to the reported case of salicylate into the LDH (Mondal et al., 2016).

#### 3.3.3 DSC

**FIGURE 5:**
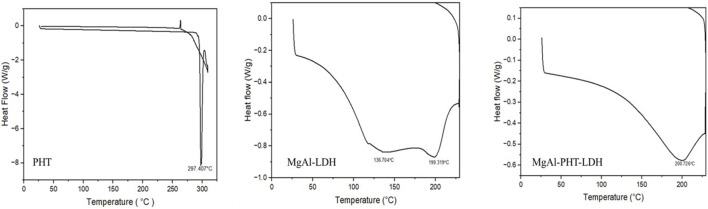
DSC thermograms of pure phenytoin, MgAl-LDH, and MgAl-PHT-LDH samples.

The calorimetric data can be the initial confirmation of another chemical entity resulting from intercalation of any molecule like drug. The DSC curves of PHT, MgAl-LDH, and MgAl-PHT-LDH are displayed in Figure 5. PHT has a distinct endothermic peak only because the drug melts at 297.407°C (Ritesh et al., 2014). The MgAl-LDH curve showed two tiny and widened peaks at 136.704°C and 199.319°C due to an early dehydroxylation of the hydroxide layers (Bini et al., 2019). For MgAl-PHT-LDH, the curve showed the melting endotherm at 200.726°C that was not present in the PHT sample and the disappearance of the peak at 136.704°C in the LDH sample. Therefore, in the DSC thermogram of MgAl-PHT-LDH, the endotherm peak at 200.726°C represents the identity of a different product than that of PHT and MgAl-LDH; confirming the successful intercalation of PHT into MgAl-LDH to form MgAl-PHT-LDH.

#### 3.3.4 TGA

**FIGURE 6:**
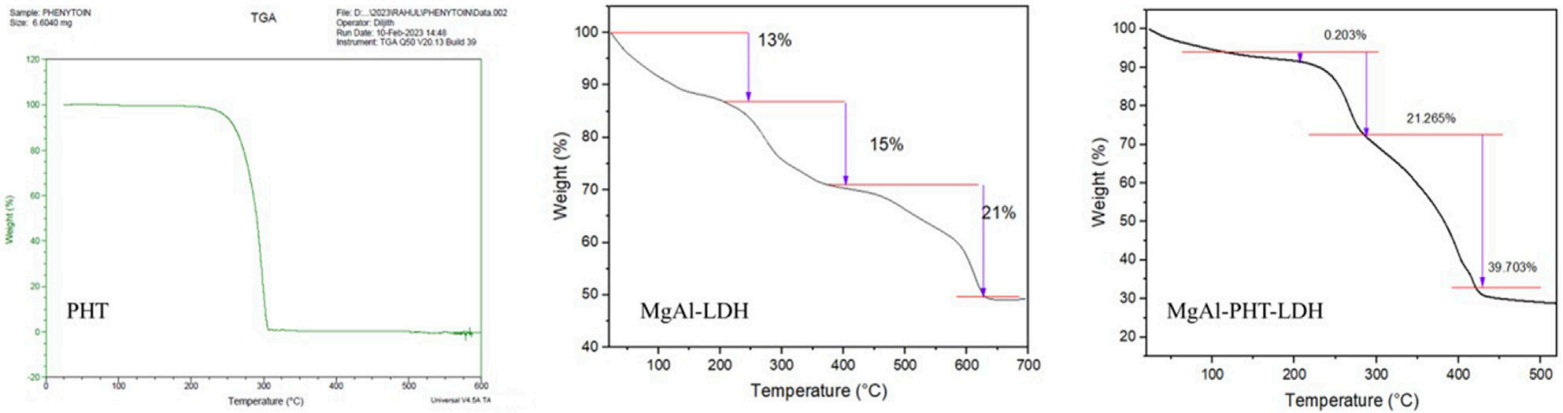
Thermogravimetric curves of pure phenytoin, MgAl-LDH, and MgAl-PHT-LDH.

Thermal analyses were done to characterize the behavior of PHT with MgAl-LDH (Figure 6). Thermogravimetric analysis of PHT revealed one step of degradation. It began at 225°C and concluded at 306°C, which could be a result of the elimination of water. Interestingly, it is reported that the losses of both adsorbed and structured waters are most likely the causes of the mass variations detected by TGA. Further, the absence of dehydroxylation of brucite layers was confirmed as no endothermic peak was there (Timóteo et al., 2019). Further, this obtained TGA curve of PHT agreed with reported data showing such observations between 340°C and 425°C (Abdollahi et al., 2016). The thermogram of MgAl-LDH powder showed a 49% loss of its mass on heating till 700°C. Two major mass loss steps happened with an increase in the temperature. The first weight loss step in the temperature region of 220°C–400°C can be related to surface water evaporation. In the following stage, in region of 400°–627°C, loss of interlayer water molecules occurs. Such an observation was made for the hydrating component of salicylate intercalated LDH too (Mondal et al., 2016). In the MgAl-PHT-LDH thermogram, two significant stages of weight loss during thermal breakdown were observed. As seen in the curve**,** the initial phase of mass loss in the region of 206°C–302°C was caused by the removal of water absorbed on the LDH’s outer surface. The second mass loss, in the region of 302°–450°C, with a total weight loss of 21%, can be attributed to the removal of hydroxyl ions in the LDH layers and the breakdown of nitrate ions. As seen in the Figure 6, the decomposition of MgAl-PHT-LDH took a higher temperature than the decomposition of the free PHT. This implies that the intercalation improved the stability of PHT in the LDH against higher temperatures. This was in accordance with the observations made during the intercalation of protocatechuic acid in MgAl-LDH (Barahuie et al., 2013).

#### 3.3.5 Particle size and PDI

**FIGURE 7:**
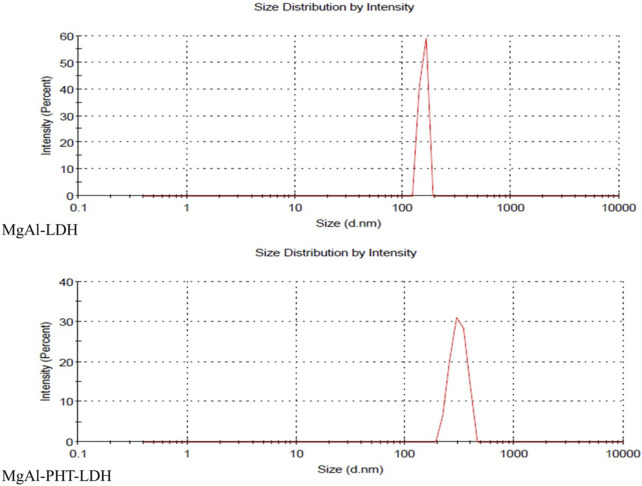
Particle size and distribution of MgAl-LDH, and MgAl-PHT-LDH.

The hydrodynamic size and PDI of MgAl-LDH were 152 ± 38.61 nm and 0.780 respectively. Meanwhile, MgAl-PHT-LDH had a size of 313 ± 109.5 nm with a PDI of 0.032 (Figure 7). The larger particle size of MgAl-PHT-LDH compared to MgAl-LDH can be attributed to the intercalation of PHT (Ladewig et al., 2010; Luengo et al., 2021). It is to be noted that the PDI value of MgAl-LDH indicated a very broad distribution of particle sizes, whereas the PDI of MgAl-PHT-LDH was very uniform. The intercalation process might have led to a more uniform distribution of the drug within the LDH layers, resulting in particles with more consistent sizes. Intercalation of the drug may also have reduced the tendency of LDH particles to aggregate or form larger agglomerates. This reduction in aggregation can result in a narrower size distribution and hence a lower PDI (Mondal et al., 2016; Pavlovic et al., 2022).

#### 3.3.6 Scanning electron microscopy (SEM)

**FIGURE 8:**
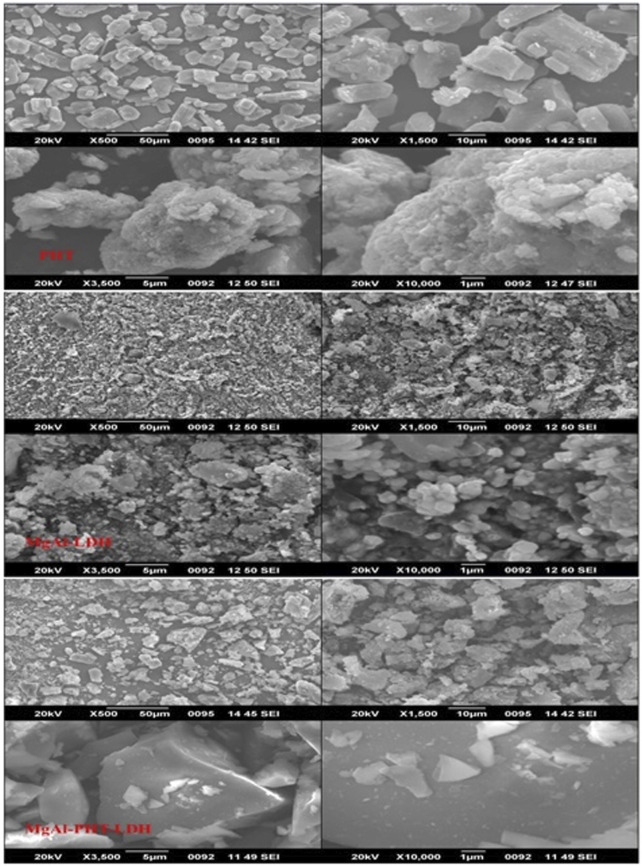
SEM images of PHT **(A–D)**, MgAl-LDH **(E–H)**, and MgAl-PHT-LDH **(I–L)**.

The morphological analysis of PHT, MgAl-LDH, and MgAl-PHT-LDH were achieved using SEM (Figure 8). For pure PHT, the SEM image clearly shows rod-like or columnar structure of the crystals, similar to the shape of PHT crystals reported by others (Nokhodchi et al., 2003). Meanwhile, MgAl-LDH showed spherical sand rose morphology as a result of the reaction between ^−^OH groups of MgAl-LDH and NaOH at a pH of 9–10. Aggregation of MgAl-LDH particles was observed, which agrees with the previously reported findings regarding Mg-Al-LDH (Beyranvand et al., 2019). Although MgAl-LDH had nearly a spherical shape, the morphology of MgAl-PHT-LDH exhibited a shape more of a square-like crystal with a wider size range. The changes observed in the crystallinity of the MgAl-LDH might be due to intercalation of PHT (Ameena Shirin et al., 2021). Furthermore, owing to the intercalation of the PHT into the LDH layers, the particle size of MgAl-PHT-LDH was larger than MgAl-LDH. These results supported the findings of particle size analysis.

#### 3.3.7 TEM

**FIGURE 9:**
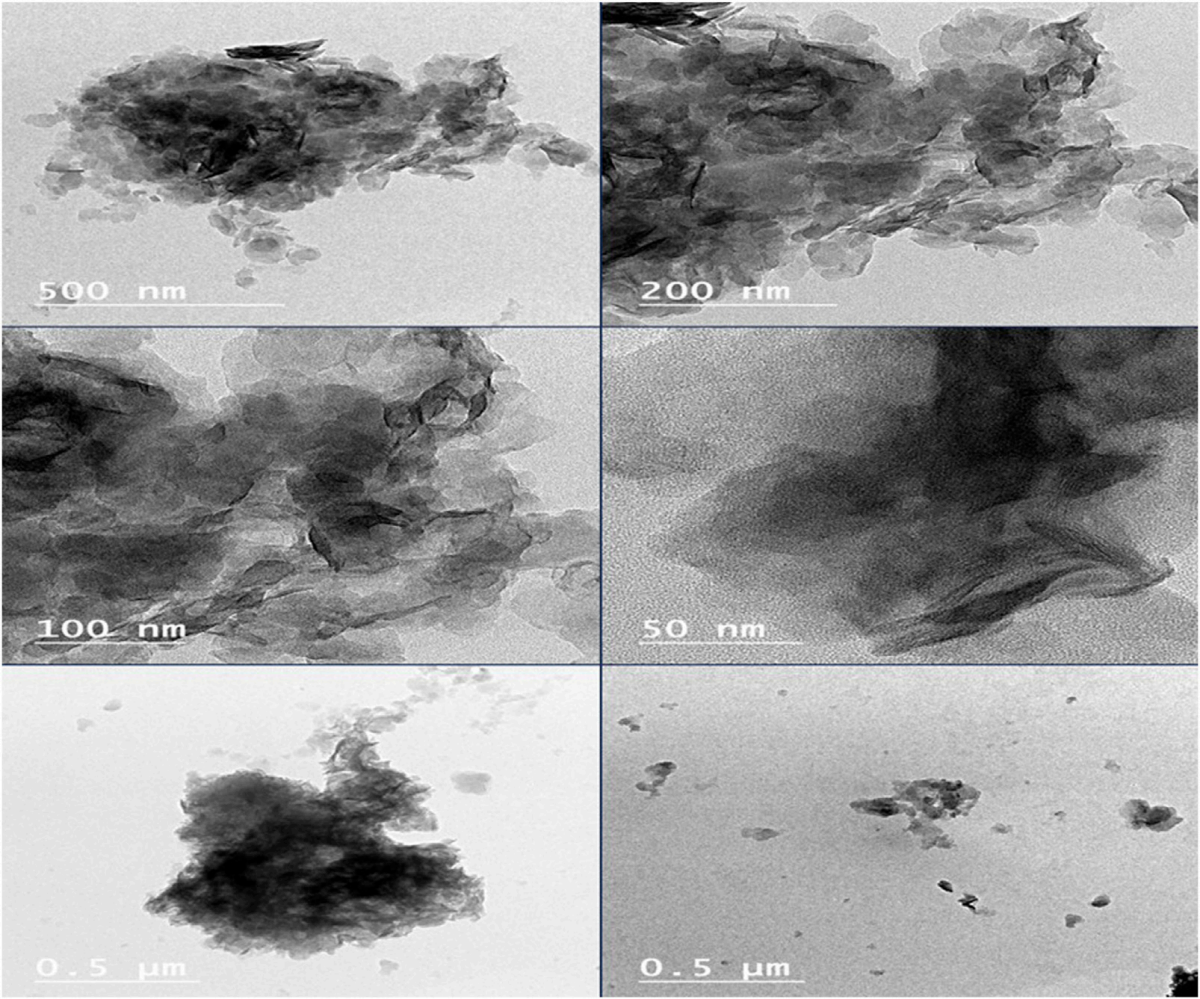
TEM images of MgAl-PHT-LDH.

The TEM micrographs of MgAl-PHT-LDH (Figure 9) exhibited morphology that was more elongated and had a wider size range. It is reported that MgAl-LDH can show a plate-like morphology in TEM images (Ouyang et al., 2022). As described in previous sections, the intercalation of PHT might have caused the conversion of the plate-like morphology to more elongated structures. The particle size of Mg-Al-PHT-LDH was high due to the intercalation of PHT into MgAl-LDH layers and confirmed the results of particle size analysis.

### 3.4 Formulation of MgAl-PHT-LDH tablets

#### 3.4.1 Preformulation studies

Table 1 displays the formula chosen for the conversion of Mg-Al-PHT-LDH into a tablet. The densities of the powder blends were measured, then Hausner’s ratio and Carr’s index were calculated. The observed pre-compression characteristics of the powder are provided in Table 3. The average angle of repose value as observed in table 3indicated an acceptable outcome. Furthermore, the Hausner ratio and Carr’s index indicated favorable flow characteristics of the powder blend in accordance with the standards outlined in the United States Pharmacopeia (USP) (Pharmacopeia, 2022).

**TABLE 3:** Pre-compression characteristics of the powder mixture.

Angle of repose (°)	Bulk density (mg/mL)	Tapped density (mg/mL)	Carr’s index (%)	Hausner ratio
31.40 ± 0.76	474.28 ± 7.65	565.52 ± 10.88	16.14	1.19

Two other formulas were prepared and tested but they did not yield satisfactory tablet properties (Supplementary Table S1). Only the formula presented in Table 1 was successfully compressed into tablets with acceptable tablet properties.

#### 3.4.2 Evaluation of MgAl-PHT-LDH compressed tablets

##### 3.4.2.1 Weight variation, thickness, hardness, friability, content uniformity, and disintegration time

Following compaction of the powder blend, the formed tablets were visually inspected, and showed no sticking or picking.

The observed post-compression characteristics are shown in Table 4. As not more than two of the prepared tablets varied from the average weight by more than 5% and none by more than 10%, the weight variation of the tablets was accepted, and they were found to comply with USP criteria (USP 41-NF 36, 2018). Tablet thickness was 4.04 ± 0.054 mm. To ensure the tablets are robust enough to endure handling and transportation, as well as to ensure proper disintegration upon ingestion, the force needed to fracture the tablet was assessed. The observed tablet hardness of 6.6 ± 0.1 kg indicated an acceptable value (dulla et al., 2018). The tablets’ fragility, measured at 0.17%, fell within the acceptable limit set by the US Pharmacopeia (USP), which mandates a value below 1%. The content uniformity of the prepared tablets of 98.81% ± 0.14% met the standards of the USP (85%–115%).

**TABLE 4:** Results of post-compression characteristics of MgAl-PHT-LDH tablets.

Weight variation (mg) Mean ± SD	Hardness (kg) Mean ± SD	Friability (%)	Thickness (mm)	Content uniformity (%)	Disintegration (min) Mean ± SD
297.7 ± 2.401	6.6 ± 0.1	0.1700	4.04 ± 0.054	98.81 ± 0.14	6.95 ± 0.31

Furthermore, the tablets disintegrated in less than 30 min, meeting the USP standard (USP 41-NF 36, 2018). The quantity of microcrystalline cellulose in the powder mixture had a notable impact on the disintegration time observed *in vitro*. Microcrystalline cellulose, a commonly employed excipient in pharmaceutical tablets, boasts strong binding properties and undergoes self-disintegration, requiring minimal lubrication (Thoorens et al., 2014). Its presence facilitates thorough blending of dry components, resulting in tablets with superior compression, minimal fragility, and high hardness (Chaerunisaa et al., 2017). Furthermore, PVP was integrated into tablet formulation due to its robust adhesion and ability to undergo plastic deformation, contributing to the production of solids with the desired hardness. Its high water solubility and low viscosity help minimize its impact on the dissolution and disintegration processes of the tablet, ensuring that the drug is released as intended (Luo et al., 2021).

##### 3.4.2.2 PHT dissolution test

**FIGURE 10:**
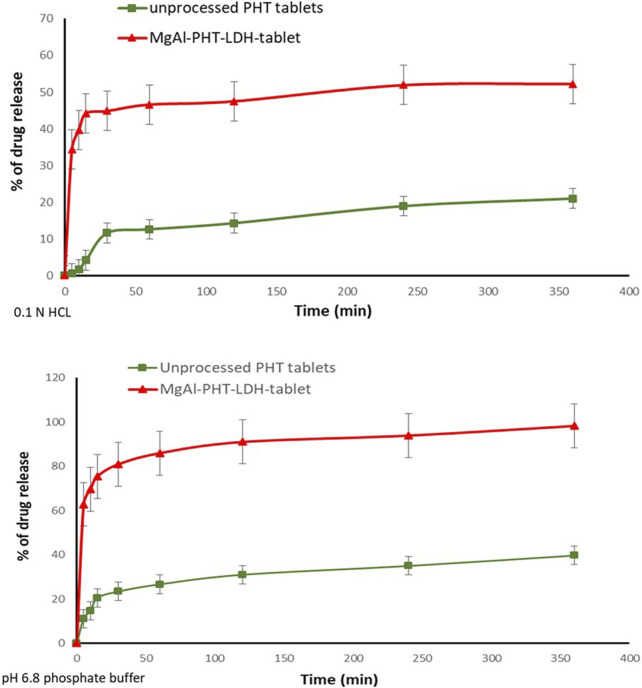
*In vitro* release of PHT from the prepared Mg-Al-LDH-PHT tablets in 0.1HCl, and pH 8.6 phosphate buffer.

The dissolution of MgAl-PHT-LDH tablets was tested both in 0.1 N HCL and pH 6.8 phosphate buffer and the dissolution profile is demonstrated in Figure 10. It was observed that the prepared tablet formulations released 44.84% and 80.89% in 0.1 N HCl and pH 6.8 phosphate buffer respectively of PHT within the first 30 min. MgAl-PHT-LDH tablets exhibited faster dissolution of PHT during the first 30 min compared to unprocessed PHT. Unprocessed PHT tablets showed a very slow and weak dissolution profile, where only less than 15% of PHT was released in the first hour in 0.1 N HCl. The f_2_ value can be applied as a useful tool to compare dissolution profiles of formulations under development or even between experimental and simulation data (Priese et al., 2023; Hu et al., 2024). The calculated f_2_ for the dissolution profiles obtained in 0.1 N HCl was 22.99 only indicating a significant difference between the dissolution profiles. The improved dissolution of MgAl-PHT-LDH could be explained by the release of PHT intercalated in the layers of LDH. Such positive effect of the LDH on dissolution has been shown previously for carprofen-intercalated LDH tablets (Capsoni et al., 2018).

The dissolution profiles of the unprocessed PHT and MgAl-PHT-LDH tablets in 6.8 phosphate buffer (Figure 10) showed that the PHT alone dissolves slowly and not more than 14% of the dose is dissolved in the first 10 min. In contrast, about 69% of PHT was released from the MgAl-PHT-LDH tablets during the same period. After 6 h, 85% of the PHT was released from MgAl-PHT-LDH tablets, unprocessed PHT tablets could release only 27% of the drug. The f_2_ for the dissolution profiles obtained in 6.8 phosphate buffer was 12.19, indicating a significant difference between the dissolution profiles observed for unprocessed PHT and MgAl-PHT-LDH tablets.

It is noteworthy to mention that the dissolution of PHT from the MgAl-PHT-LDH tablets was better in the phosphate buffer solution than that of 0.1 N HCl by comparing the amount released of drug at the same time points. As phenytoin is considered a weak acid its neutralization in alkali media becomes easier. Phenytoin’s intercalation was especially effective in ensuring that all drug doses that became available for absorption would dissolve completely and quickly.

##### 3.4.2.3 *In vivo* study

The linear regression analysis for the calibration curve and sensitivity data of PHT in rat serum using the UPLC-ESI-MS/MS approach had the following validation parameters. Calibration range (ng mL^−1^) at 10–2000, Calibration equation Y = 0.0356 X - 0.703, Slope (±SD) 0.0356 ± 0.005, Intercept (±SD) 0.703 ± 0.092, Determination coefficient (*r* ^2^) 0.9984, Correlation coefficient (r) 0.9992, Lower limit of quantification (LLOQ) (ng mL^−1^) 10, and Limit of detection (LOD) (ng mL^−1^) = 3.3. Further details are provided under Supplementary Table S2 and Supplementary Figure S1.

**FIGURE 11:**
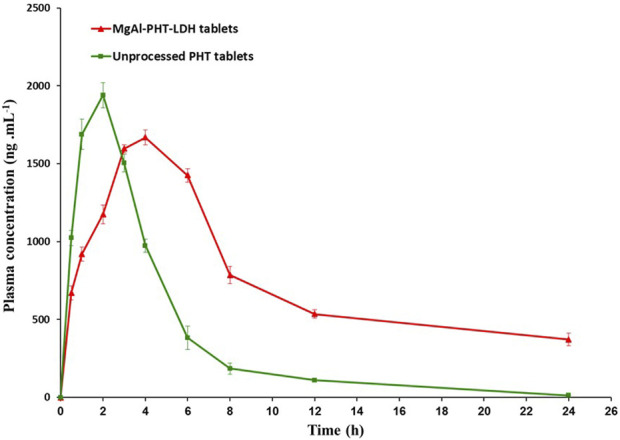
Serum concentration-time profile for LDH-phenytoin and unprocessed phenytoin in rat serum after oral administration (30 mg/kg).

After a single oral administration of 30 mg/kg PHT to rats, the serum concentration-time profile from the MgAl-PHT-LDH tablets was compared to that from unprocessed PHT tablets as represented in Figure 11.

Serum concentration of PHT was significantly higher in animal group administering MgAl-PHT-LDH compared to those receiving unprocessed PHT at all time points (*P* < 0.05). The computed values of the pharmacokinetic parameters for PHT are listed in Table 5. The results indicated longer T_max_ (4.0 ± 1.8 h) and lower C_max_ (1669.0 ± 46.9 ng.mL^−1^) values of MgAl-PHT-LDH tablets compared to unprocessed PHT tablets. The intercalation of PHT to the MgAl-LDH could be the reason for a longer T_max_ observed for MgAl-PHT-LDH tablets. Such an observation of delaying T_max_ and reduction in C_max_ was reported previously for acetylsalicylic acid when intercalated in LDH (Dong et al., 2013). However, in comparison to unprocessed PHT tablets, MgAl-PHT-LDH tablets exhibited a significantly higher area under the curve (AUC) as compared to unprocessed PHT tablets, which indicates a significant increase in relative bioavailability. The MgAl-PHT-LDH tablets showed an AUC_0-24_ of 9,936.4 ± 96.4 ng.h mL^−1^ whereas the unprocessed PHT tablets showed 7634.3 ± 58.3 ng.h mL^−1^ only. Thus, the MgAl-PHT-LDH tablets demonstrated a relative bioavailability of 130.15% in comparison to the unprocessed PHT tablets.

**TABLE 5:** Pharmacokinetic parameters of PHT in rat serum after oral administration of MgAl-PHT-LDH tablets and unprocessed PHT tablets at a PHT dose of 30 mg/kg.

Parameter	Unit	MgAl-PHT-LDH tablets		Unprocessed PHT tablets		*P*-Value (Inference)
		Average	SD	Average	SD	
C_max_	ng.mL^-1^	1669.0	46.9	1939.3	81.9	*P* < 0.05 (significant difference)
T_max_	h	4.0	1.8	2.0	0.9	*P* > 0.05 (no significant difference)
AUC_0-t4_	ng.h mL^-1^	9,936.4	96.4	7634.3	58.3	*P* < 0.05 (significant difference)
AUC_0-∞_	ng.h mL^-1^	13631.5	719.7	8635.0	80.6	*P* < 0.05 (significant difference)
AUMC_0-24_	ng.h mL^-1^	41401.3	2148.2	21836.4	1076.9	*P* < 0.05 (significant difference)
AUMC_0-∞_	ng.h mL^-1^	118682.3	6716.4	59843.1	3483.2	*P* < 0.05 (significant difference)
MRT	h	8.7	0.39	3.86	0.19	*P* < 0.05 (significant difference)
T_1/2_	h	6.03	0.53	2.67	0.12	*P* < 0.05 (significant difference)
CL	L kg^-1^ h^-1^	3.02	0.26	3.929	0.21	*P* < 0.05 (significant difference)

The findings of the *in vivo* experiment demonstrated that the intercalation of PHT in MgAl-LDH increased the rate and extent of absorption, and thereby improved the oral bioavailability of PHT. These findings were in agreement with reported observations for other drugs after intercalation in LDH (Capsoni et al., 2018; Bini et al., 2019; Leão et al., 2019).

##### 3.4.2.4 Accelerated stability studies

For stability study, tablets were investigated after keeping in a desiccator for 6 months as mentioned in the “Methods” section. The results of weight variation, content uniformity, hardness, thickness, friability, and *in vitro* disintegration time are presented in Table 6.

**TABLE 6:** Results of accelerated stability studies.

Weight variation (mg) Mean ± SD		Hardness (kg) Mean ± SD		Friability (%)		Thickness (mm)		Content uniformity (%)		Disintegration (min) Mean ± SD	
Initial	6 months	Initial	6 months	Initial	6 months	Initial	6 months	Initial	6 months	Initial	6 months
297.7 ± 2.401	296.1 ± 1.70	6.6 ± 0.1	6.56 ± 0.05	0.1700	0.1700	4.04 ± 0.054	4.06 ± 0.057	98.81 ± 0.14	98.34 ± 0.21	6.95 ± 0.31	6.89 ± 0.18

*P* > 0.05 (no significant difference).

## 4 Conclusion

This study aimed to investigate the enhancement of solubility, dissolution, and bioavailability of PHT, after its intercalation to MgAl-PHT-LDH. Based on the findings of the current study, intercalation of PHT to MgAl-PHT-LDH could be achieved successfully as confirmed by FTIR, PXRD, DSC, and TGA studies. This intercalation effectively improved drug solubility in 0.1N HCl and phosphate buffer (pH6.8). MgAl-PHT-LDH along with the selected excipients could be formulated into tablets that showed acceptable pre-compression and tablet properties. Finally, the study was capable of proofing enhanced *in vivo* bioavailability of intercalated PHT compared to unprocessed PHT at all time points in rat animal model. Overall, MgAl-PHT-LDH tablet could be considered as a potential candidate for clinical evaluation in the treatment of seizures at lower doses of PHT.

## Data Availability

The original contributions presented in the study are included in the article/Supplementary Material, further inquiries can be directed to the corresponding author.

## References

[B1] Abdollahi E. Abdouss M. Mohammadi A. (2016). Synthesis of a nano molecularly imprinted polymeric sorbent for solid phase extraction and determination of phenytoin in plasma, urine, and wastewater by HPLC. RSC Adv. 6, 39095–39105. 10.1039/c6ra00421k

[B2] Alqahtani M. S. Kazi M. Alsenaidy M. A. Ahmad M. Z. (2021). Advances in oral drug delivery. Front. Pharmacol. 12, 618411. 10.3389/fphar.2021.618411 33679401 PMC7933596

[B3] Ameena Shirin V. K. Sankar R. Johnson A. P. Gangadharappa H. V. Pramod K. (2021). Advanced drug delivery applications of layered double hydroxide. J. Control. Release 330, 398–426. 10.1016/j.jconrel.2020.12.041 33383094

[B4] Ay A. N. Zümreoglu-Karan B. Mafra L. (2009). A simple mechanochemical route to layered double hydroxides: synthesis of hydrotalcite-like Mg-Al-NO3-LDH by manual grinding in a mortar. Z Anorg. Allg. Chem. 635, 1470–1475. 10.1002/zaac.200801287

[B5] Barahuie F. Hussein M. Z. Hussein-Al-Ali S. H. Arulselvan P. Fakurazi S. Zainal Z. (2013). Preparation and controlled-release studies of a protocatechuic acid-magnesium/aluminumlayered double hydroxide nanocomposite. Int. J. Nanomedicine 8, 1975–1987. 10.2147/IJN.S42718 23737666 PMC3669093

[B6] Berber M. R. Minagawa K. Katoh M. Mori T. Tanaka M. (2008). Nanocomposites of 2-arylpropionic acid drugs based on Mg–Al layered double hydroxide for dissolution enhancement. Eur. J. Pharm. Sci. 35, 354–360. 10.1016/j.ejps.2008.08.006 18789388

[B7] Beyranvand N. S. Samiey B. Tehrani A. D. (2019). Adsorption mechanism of Congo red on Mg–Al-layered double hydroxide nanocompound. Acta Chim. Slov. 66, 443–454. 10.17344/acsi.2018.4920 33855491

[B8] Bi X. Zhang H. Dou L. (2014). Layered double hydroxide-based nanocarriers for drug delivery. Pharmaceutics 6, 298–332. 10.3390/pharmaceutics6020298 24940733 PMC4085601

[B9] Bini M. Monteforte F. Quinzeni I. Friuli V. Maggi L. Bruni G. (2019). Hybrid compounds for improving drugs solubility: synthesis, physico-chemical and pharmaceutical characterization of Nimesulide-LDH. J. Solid State Chem. 272, 131–137. 10.1016/j.jssc.2019.02.001

[B10] Browne T. R. LeDuc B. (1995). “Phenytoin: chemistry and biotransformation,” in Antiepileptic drugs. Editors Levy R. H. Mattson R. H. Meldrum B. S. fourth ed. (New York: Raven Press), 283–300.

[B11] Burstein A. H. Cox D. S. Mistry B. Eddington N. D. (1999). Phenytoin pharmacokinetics following oral administration of phenytoin suspension and fosphenytoin solution to rats. Epilepsy Res. 34, 129–133. 10.1016/S0920-1211(98)00107-7 10210027

[B12] Capsoni D. Quinzeni I. Bruni G. Friuli V. Maggi L. Bini M. (2018). Improving the carprofen solubility: synthesis of the Zn2Al-LDH hybrid compound. J. Pharm. Sci. 107, 267–272. 10.1016/j.xphs.2017.09.019 28987499

[B13] Chaerunisaa A. Y. Sriwidodo S. Abdassah M. (2017). MCCs as pharmaceutical excipient.

[B14] del Arco M. Fernández A. Martín C. Rives V. (2010). Solubility and release of fenbufen intercalated in Mg, Al and Mg, Al, Fe layered double hydroxides (LDH): the effect of Eudragit® S 100 covering. J. Solid State Chem. 183, 3002–3009. 10.1016/j.jssc.2010.10.017

[B15] de Oliveira E. G. Cardoso A. M. Paese K. Coradini K. de Oliveira C. V. Pohlmann A. R. (2018). Reconstituted spray-dried phenytoin-loaded nanocapsules improve the *in vivo* phenytoin anticonvulsant effect and the survival time in mice. Int. J. Pharm. 551, 121–132. 10.1016/j.ijpharm.2018.09.023 30218826

[B16] Dobrucki R. Wojciechowska A. (1992). Micronization of phenytoin by precipitation. Acta Pol. Pharm. 49, 103–107. 8769084

[B17] Dong L. Gou G. Jiao L. (2013). Characterization of a dextran–coated layered double hydroxide acetylsalicylic acid delivery system and its pharmacokinetics in rabbit. Acta Pharm. Sin. B 3, 400–407. 10.1016/j.apsb.2013.09.003

[B18] dulla O. Sultana S. Shohag Hosen M. (2018). *In vitro* comparative quality evaluation of different brands of esomeprazole tablets available in selected community pharmacies in Dhaka, Bangladesh. BMC Res. Notes 11, 184. 10.1186/s13104-018-3285-x 29554952 PMC5859528

[B19] Hu J. Wan J. Xi J. Shi W. Qian H. (2024). AI-driven design of customized 3D-printed multi-layer capsules with controlled drug release profiles for personalized medicine. Int. J. Pharm. 656, 124114. 10.1016/j.ijpharm.2024.124114 38615804

[B20] Hua S. (2020). Advances in oral drug delivery for regional targeting in the gastrointestinal tract - influence of physiological, pathophysiological and pharmaceutical factors. Front. Pharmacol. 11, 524. 10.3389/fphar.2020.00524 32425781 PMC7212533

[B21] Khan K. U. Minhas M. U. Badshah S. F. Suhail M. Ahmad A. Ijaz S. (2022). Overview of nanoparticulate strategies for solubility enhancement of poorly soluble drugs. Life Sci. 291, 120301. 10.1016/j.lfs.2022.120301 34999114

[B22] Khusnutdinov V. R. Isupov V. P. (2019). Mechanochemical synthesis of nanocomposites based on Fe3O4 and layered double hydroxides. Mater Today Proc. 12, 48–51. 10.1016/j.matpr.2019.03.061

[B23] Ladewig K. Niebert M. Xu Z. P. Gray P. P. Lu G. Q. (2010). Controlled preparation of layered double hydroxide nanoparticles and their application as gene delivery vehicles. Appl. Clay Sci. 48, 280–289. 10.1016/j.clay.2009.11.032

[B24] Latrofa A. Trapani G. Franco M. Serra M. Muggironi M. Fanizzi F. P. (2001). Complexation of phenytoin with some hydrophilic cyclodextrins: effect on aqueous solubility, dissolution rate, and anticonvulsant activity in mice. Eur. J. Pharm. Biopharm. 52, 65–73. 10.1016/s0939-6411(01)00144-8 11438425

[B25] Leão A. D. Oliveira V. V. Marinho F. A. Wanderley A. G. Aguiar J. S. Silva T. G. (2019). Hybrid systems of glibenclamide and layered double hydroxides for solubility enhancement for the treatment of diabetes mellitus II. Appl. Clay Sci. 181, 105218. 10.1016/j.clay.2019.105218

[B26] Luengo C. V. Crescitelli M. C. Lopez N. A. Avena M. J. (2021). Synthesis of layered double hydroxides intercalated with drugs for controlled release: successful intercalation of ibuprofen and failed intercalation of paracetamol. J. Pharm. Sci. 110, 1779–1787. 10.1016/j.xphs.2021.01.023 33513404

[B27] Luo Y. Hong Y. Shen L. Wu F. Lin X. (2021). Multifunctional role of Polyvinylpyrrolidone in pharmaceutical formulations. AAPS PharmSciTech 22, 34. 10.1208/s12249-020-01909-4 33404984

[B28] Maggi L. Bruni G. Ferrara C. Puscalau C. Quinzeni I. Friuli V. (2023). Zaltoprofen-layered double hydroxide hybrids to enhance zaltoprofen solubility and dissolution rate. Appl. Clay Sci. 231, 106723. 10.1016/j.clay.2022.106723

[B29] Mishra G. Dash B. Pandey S. (2018). Layered double hydroxides: a brief review from fundamentals to application as evolving biomaterials. Appl. Clay Sci. 153, 172–186. 10.1016/j.clay.2017.12.021

[B30] Modi D. Jonnalagadda S. Campbell G. A. Dalwadi G. (2023). Enhancing oil solubility of BCS class II drug phenytoin through hydrophobic ion pairing to enable high drug load in injectable nanoemulsion to prevent precipitation at physiological pH with a potential to prevent phlebitis. J. Pharm. Sci. 112, 2427–2443. 10.1016/j.xphs.2023.03.012 36958691

[B31] Mondal S. Dasgupta S. Maji K. (2016). MgAl- layered double hydroxide nanoparticles for controlled release of salicylate. Mater. Sci. Eng. C 68, 557–564. 10.1016/j.msec.2016.06.029 27524054

[B32] Moore J. W. Flanner H. H. (1996). Mathematical comparison of dissolution profiles. Pharm. Technol. 20, 64–74.

[B33] Nokhodchi A. Bolourtchian N. Dinarvand R. (2003). Crystal modification of phenytoin using different solvents and crystallization conditions. Int. J. Pharm. 250, 85–97. 10.1016/S0378-5173(02)00488-X 12480275

[B34] Ouyang Y. Li L.-X. Xie Z.-H. Tang L. Wang F. Zhong C.-J. (2022). A self-healing coating based on facile pH-responsive nanocontainers for corrosion protection of magnesium alloy. J. Magnesium Alloys 10, 836–849. 10.1016/j.jma.2020.11.007

[B35] Pade V. Stavchansky S. (1998). Link between drug absorption solubility and permeability measurements in Caco-2 cells. J. Pharm. Sci. 87, 1604–1607. 10.1021/js980111k 10189274

[B36] Patocka J. Wu Q. Nepovimova E. Kuca K. (2020). Phenytoin – an anti-seizure drug: overview of its chemistry, pharmacology and toxicology. Food Chem. Toxicol. 142, 111393. 10.1016/j.fct.2020.111393 32376339

[B37] Pavlovic M. Szerlauth A. Muráth S. Varga G. Szilagyi I. (2022). Surface modification of two-dimensional layered double hydroxide nanoparticles with biopolymers for biomedical applications. Adv. Drug Deliv. Rev. 191, 114590. 10.1016/j.addr.2022.114590 36341860

[B38] Pharmacopeia (2022). General chapters: <1174> POWDER FLOW. Available at: http://www.pharmacopeia.cn/v29240/usp29nf24s0_c1174.html (Accessed November 14, 2022).

[B39] Priese F. Wiegel D. Funaro C. Mondelli G. Wolf B. (2023). Comparison of mini-tablets and pellets as multiparticulate drug delivery systems for controlled drug release. Coatings 13, 1891. 10.3390/coatings13111891

[B40] Ramadhan U. H. (2012). The synthesis of new phenytoin derivative and the study of its inhibition activity to cyclooxygenase-2 (COX-2). J. Basrah Res. 2.

[B41] Ritesh P. Shivshankar M. Ashish W. Mahesh N. Vinayak S. (2014). Assessment of effects of extract from roots and leaves of citrullus lanatus, (Thunb). J. Med. Chem. Drug Discov. 5, 33–42.

[B42] Serajuddin A. T. M. Jarowski C. I. (1993). Influence of pH on release of phenytoin sodium from slow-release dosage forms. J. Pharm. Sci. 82, 306–310. 10.1002/jps.2600820318 8450427

[B43] Sheir M. M. Nasra M. M. A. Abdallah O. Y. (2022). Phenytoin-loaded bioactive nanoparticles for the treatment of diabetic pressure ulcers: formulation and *in vitro*/*in vivo* evaluation. Drug Deliv. Transl. Res. 12, 2936–2949. 10.1007/s13346-022-01156-z 35403947 PMC9636106

[B44] Stavchansky S. Gowan W. G. (1984). Evaluation of the bioavailability of a solid dispersion of phenytoin in polyethylene glycol 6000 and a commercial phenytoin sodium capsule in the dog. J. Pharm. Sci. 73, 733–736. 10.1002/jps.2600730607 6376769

[B45] Suneetha S. C. A. Raghupathy B. P. C. Suresh P. K. (2014). Physicochemical characterization and cytotoxicity screening of a novel colloidal nanogold-based phenytoin conjugate. Sci. Pharm. 82, 857–872. 10.3797/scipharm.1402-03 26171330 PMC4475805

[B46] Thoorens G. Krier F. Leclercq B. Carlin B. Evrard B. (2014). Microcrystalline cellulose, a direct compression binder in a quality by design environment—a review. Int. J. Pharm. 473, 64–72. 10.1016/j.ijpharm.2014.06.055 24993785

[B47] Timóteo T. R. R. de Melo C. G. Danda L. J. D. A. Silva L. C. P. B. B. Fontes D. A. F. Silva P. C. D. (2019). Layered double hydroxides of CaAl: a promising drug delivery system for increased dissolution rate and thermal stability of praziquantel. Appl. Clay Sci. 180, 105197. 10.1016/j.clay.2019.105197

[B48] USP 41-NF 36 (2018). The United States Pharmacopeia national formulary.

[B49] Widanapathirana L. Tale S. Reineke T. M. (2015). Dissolution and solubility enhancement of the highly lipophilic drug phenytoin via interaction with poly(N-isopropylacrylamide-co-vinylpyrrolidone) excipients. Mol. Pharm. 12, 2537–2543. 10.1021/acs.molpharmaceut.5b00202 26046484

[B50] Yasir M. Asif M. Kumar A. Aggarval A. (2010). Biopharmaceutical classification system: an account. Int. J. Pharmtech Res. 2, 1681–1690.

[B51] Yu S. Choi G. Choy J.-H. (2023). Multifunctional layered double hydroxides for drug delivery and imaging. Nanomater. (Basel) 13, 1102. 10.3390/nano13061102 PMC10058705 36985996

