# Supplementary material for: Improvement of solubility, dissolution, and bioavailability of phenytoin intercalated in Mg-Al layered double hydroxide
Source: Front Pharmacol. 2024 Aug 2;15:1440361. doi: 10.3389/fphar.2024.1440361 (PMC11327046; doi:10.3389/fphar.2024.1440361)
Supplement: Supplementary file 1 [file DataSheet1.docx]

Supplementary Material

**Intercalation of phenytoin in Mg-Al layered double hydroxide enhanced its solubility, dissolution, and bioavailability**

# Supplementary Data

Table S1: Quantity of each excipient and the result of tablet-breaking and in vitro disintegration

| Run | Mg-Al-PHT-LDH (mg) | PVP (mg) | MCC (mg) | Talc (mg) | Mg-stearate (mg) | Starch (mg) | Lactose (mg) | Tablet breaking force (kg) | In-vitro disintegration (min) |
| --- | --- | --- | --- | --- | --- | --- | --- | --- | --- |
| F1 | 200 | 9 | 60 | 6 | 3 | - | 22 | 8.06 ± 0.15 | 9.32 ± 0.3 |
| F2 | 200 | 9 | 60 | 6 | 3 | 22 | - | 7.1 ± 0.1 | 7.50 ± 0.06 |
| F3 | 200 | 9 | 60 | 1.5 | 1.5 | 28 | - | 6.7 ± 0.15 | 6.76 ± 0.36 |

Tablet hardness and *in vitro* disintegration were conducted to choose the formulation with the best quality characteristics. Following compression, the excipient concentrations that produced the best tablet-breaking force were chosen. The optimized tablet formulation was based on these concentration levels. Table S1 summarizes the quantity of each excipient and the result of tablet-breaking force and *in vitro* disintegration.

In the F1 formulation in which lactose was used as filling excipient tablets was too hard as the tablet breaking force and *in vitro* disintegration time was too high. In F2, wherein starch was used instead of lactose, tablet breaking force and *in vitro* disintegration time were in the acceptable range, but tablets come with some deformity. In F3 we reduced the proportion of Talc and Mg-stearate from 2 % and 1% respectively to 0.5 %, to avoid affecting tablet dissolution. So we got a well-formed tablet with a good result of tablet breaking force and *in vitro* disintegration time. Therefore, F3 was selected as the optimized formula because it showed better results.

**Utilizing Ultra-Performance Liquid Chromatography-Electrospray Ionization-Tandem Mass Spectrometry (UPLC-ESI-MS/MS)**

This analytical configuration comprised an Agilent UPLC system, incorporating a 1260 Infinity II quaternary pump with an integrated degassing unit, a column thermostat, and a 1260 Infinity II autosampler, all sourced from Agilent. Nitrogen gas was provided by the NG CASTORE XS iQ nitrogen generator from LNI Swiss gas located in Versoix, Switzerland. Chromatographic separation was achieved using a reversed-phase C18-Eclipse-Plus-RRHD column (50 × 2.1 mm, 1.8 μm) from Agilent. The mobile phase, consisting of a 50:50 (v/v) mixture of water (containing 0.1% formic acid) and acetonitrile (containing 0.1% formic acid), flowed at a rate of 0.4 mL min^-1^, while the column temperature was maintained at 40°C. Prior to analysis, the column was equilibrated with the mobile phase. The UPLC system was linked to an Ultivo triple quadrupole mass spectrometer produced by Agilent Technologies. The mass spectrometer operated in positive electrospray ionization (ESI) mode, employing multiple reaction monitoring (MRM) for PHT detection. Data processing was carried out using Mass Hunter Quantitative Data Analysis software. Specific MRM transitions were selected for quantification based on the most intense fragment ion, with a mass-to-charge ratio (m/z) of 253.1→182.2 for PHT. Each MRM transition was set to a dwell time of 25 ms. The parameters of the ESI Jet Stream source were fine-tuned to enhance the MRM signals, including a gas temperature of 300°C, a gas flow rate of 7 L/min, a nebulizer gas pressure of 15 psi, a capillary voltage of 4000 V, a fragmentor voltage of 36 V, and a collision energy (CE) of 41 V.

**Table S2:** The linear regression analyses for the calibration curve and sensitivity data of Phenytoin in rat plasma using UPLC-ESI-MS/MS approach.

| Validation parameter | Phenytoin regression data |
| --- | --- |
| Linearity and range ^a, b^  Calibration range (ng mL^-1^) | 10 - 2000 |
| Calibration equation  Slope (±SD) | Y = 0.0356 X - 0.703  0.0356 (±0.005) |
| Intercept (±SD) | 0.703 (±0.092) |
| Determination coefficient (r^2^)  Correlation coefficient (r) | 0.9984  0.9992 |
| **Sensitivity**  LLOQ (ng mL^-1^)  LOD | 10  3.3 |

^a^ Peak area ratio of the Phenytoin/ I.S. against concentrations (ng mL^-1^).

^b^ Data presented as mean (n=3) ± SD.

**Supplementary Figure 1.**

Figure S1 : Calibration curve of Phenytoin in rat plasma.

**
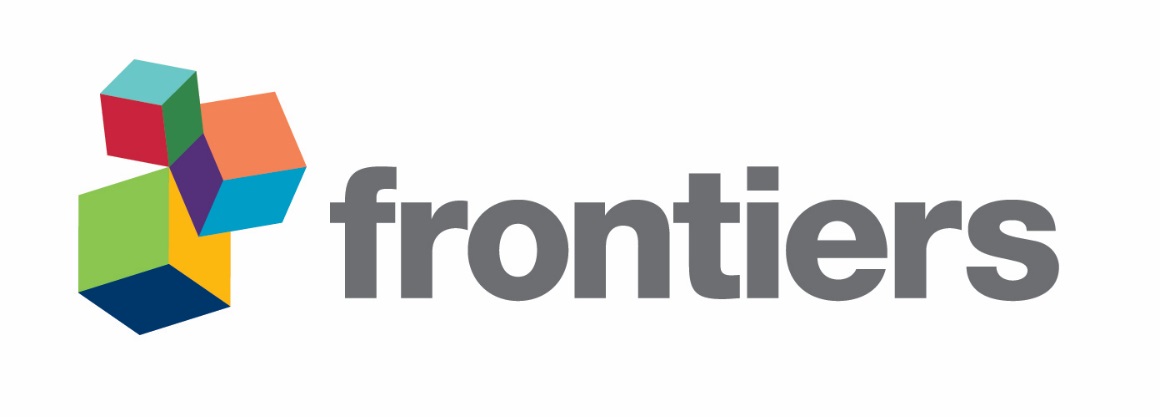
**
